# Substantial asymptomatic submicroscopic *Plasmodium* carriage during dry season in low transmission areas in Senegal: Implications for malaria control and elimination

**DOI:** 10.1371/journal.pone.0182189

**Published:** 2017-08-03

**Authors:** Makhtar Niang, Laty Gaye Thiam, Rokhaya Sane, Nafissatou Diagne, Cheikh Talla, Souleymane Doucoure, Joseph Faye, Fode Diop, Abdoulaye Badiane, Babacar Diouf, Diogop Camara, Fatoumata Diene-Sarr, Cheikh Sokhna, Vincent Richard, Aissatou Toure-Balde

**Affiliations:** 1 Immunology Unit, Institut Pasteur Dakar, Dakar, Sénégal; 2 French National Research Institute for Sustainable Development, URMITE, URMITE UMR 198, Dakar, Sénégal; 3 Epidemiology Unit, Institut Pasteur Dakar, Dakar, Sénégal; Université Pierre et Marie Curie, FRANCE

## Abstract

**Background:**

In the progress towards malaria elimination, the accurate diagnosis of low-density asymptomatic infections is critical. Low-density asymptomatic submicroscopic malaria infections may act as silent reservoirs that maintain low-level residual malaria transmission in the community. Light microscopy, the gold standard in malaria diagnosis lacks the sensitivity to detect low-level parasitaemia. In this study, the presence and prevalence of submicroscopic *Plasmodium* carriage were investigated to estimate the parasites reservoir among asymptomatic individuals living in low transmission areas in Dielmo and Ndiop, Senegal during the dry season.

**Methods:**

A total of 2,037 blood samples were collected during cross-sectional surveys prior the malaria transmission season in July 2013 (N = 612), June 2014 (N = 723) and June 2015 (N = 702) from asymptomatic individuals living in Dielmo and Ndiop, Senegal. Samples were used to determine the prevalence of submicroscopic *Plasmodium* carriage by real time PCR (qPCR) in comparison to microscopy considered as gold standard.

**Results:**

The prevalence of submicroscopic *Plasmodium* carriage was 3.75% (23/612), 12.44% (90/723) and 6.41% (45/702) in 2013, 2014 and 2015, respectively. No *Plasmodium* carriage was detected by microscopy in 2013 while microscopy-based prevalence of *Plasmodium* carriage accounted for only 0.27% (2/723) and 0.14% (1/702) in 2014 and 2015, respectively. *Plasmodium falciparum* accounted for the majority of submicroscopic infections and represented 86.95% (20/23), 81.11% (73/90) and 95.55 (43/45) of infections in 2013, 2014 and 2015 respectively.

**Conclusion:**

Low-density submicroscopic asymptomatic *Plasmodium* carriage is common in the study areas during the dry season indicating that traditional measures are insufficient to assess the scale of parasite reservoir when transmission reaches very low level. Control and elimination strategies may wish to consider using molecular methods to identify parasites carriers to guide Mass screening and Treatment strategies.

## Introduction

Malaria due to *Plasmodium falciparum* remains a significant public health problem despite largely successful control efforts that have led to a sharp decrease of malaria transmission, morbidity and mortality in recent years [1]. According to World Health Organization (WHO), malaria mortality rates fell by 47% worldwide and by 54% in sub-Saharan Africa between 2000 and 2013 [1]. This success has mostly been attributed to coordinated scale-up of global malaria preventive and control interventions.

Most malaria surveillance systems, including the system in Senegal, are predicated on detection of symptomatic malaria cases at health facilities using either clinical diagnosis alone or with parasitological confirmation by light microscopy (LM) or rapid diagnostic tests (RDT). In most settings, where the goal is to significantly reduce malaria morbidity and mortality, quality assured LM and RDT have been shown to effectively detect parasites in the majority of symptomatic patients and thus guide treatments. In contrast, as malaria transmission declines and countries progress towards elimination, LM and RDT are insufficiently sensitive to detect low-level parasitaemia, thus causing gross underestimation of parasite prevalence in areas where most infections are subpatent [2–6].

Although LM remains the gold standard for the diagnosis of malaria and quantification of *Plasmodium* parasites, the rapid advances in molecular biology and nucleic acid testing methods and their routine application in clinical studies and epidemiological surveys have enabled the detection of low-density submicroscopic infections as reported in several studies [7–10]. Moreover, the development of highly sensitive, specific and quantitative molecular diagnostic tests for malaria are becoming increasingly important as control strategies seek to eliminate asymptomatic infections that serve as reservoirs for transmission [11, 12].

To date, the importance of sub-microscopic infections for malaria transmission is still unclear [13]. However, according to the Malaria Eradication Research Agenda, any parasitaemia regardless of its size might contribute to transmission and threaten malaria elimination efforts [11]. Therefore, more sensitive diagnostics are required to accurately document the extent and distribution of low-density submicroscopic hidden malaria infections in local communities as they may represent reservoirs of parasite capable of effectively sustaining transmission to mosquitoes when conditions become optimal [14, 15].

Cross-sectional surveys that sample from asymptomatic individuals provide an operationally attractive method to estimate the prevalence of *Plasmodium* parasites carriage in the wider catchment population; therefore mitigating against some of the biases associated with passive case detection [16, 17].

In Dielmo and Ndiop, two Senegalese villages where longitudinal studies on determinants of malaria infections have been conducted since early 1990s [18], the disease epidemiology has since changed greatly [19, 20]. *P*. *falciparum* prevalence in asymptomatic individuals estimated by LM has been shown to decline from 85% to 1% in 0–3 years old children and 34% to 2% in adults > 50 years between 1990 and 2010 [21].

The present study sought to determine the prevalence of submicroscopic *Plasmodium* spp parasites carriage in community-based cross sectional surveys conducted prior the malaria transmission season in two Senegalese villages, Dielmo and Ndiop, where all malaria indicators have declined substantially over the last decade.

## Materials and methods

### Study sites and samples collection

The study was conducted in Dielmo and Ndiop, two extensively described malaria endemic villages situated in Fatick region, Senegal [18, 22]. In both villages, the malaria disease epidemiology has greatly changed during the last decade with a substantial decrease of all malaria indicators (transmission intensity, parasite prevalence, morbidity and mortality) [20].

**Fig 1:**
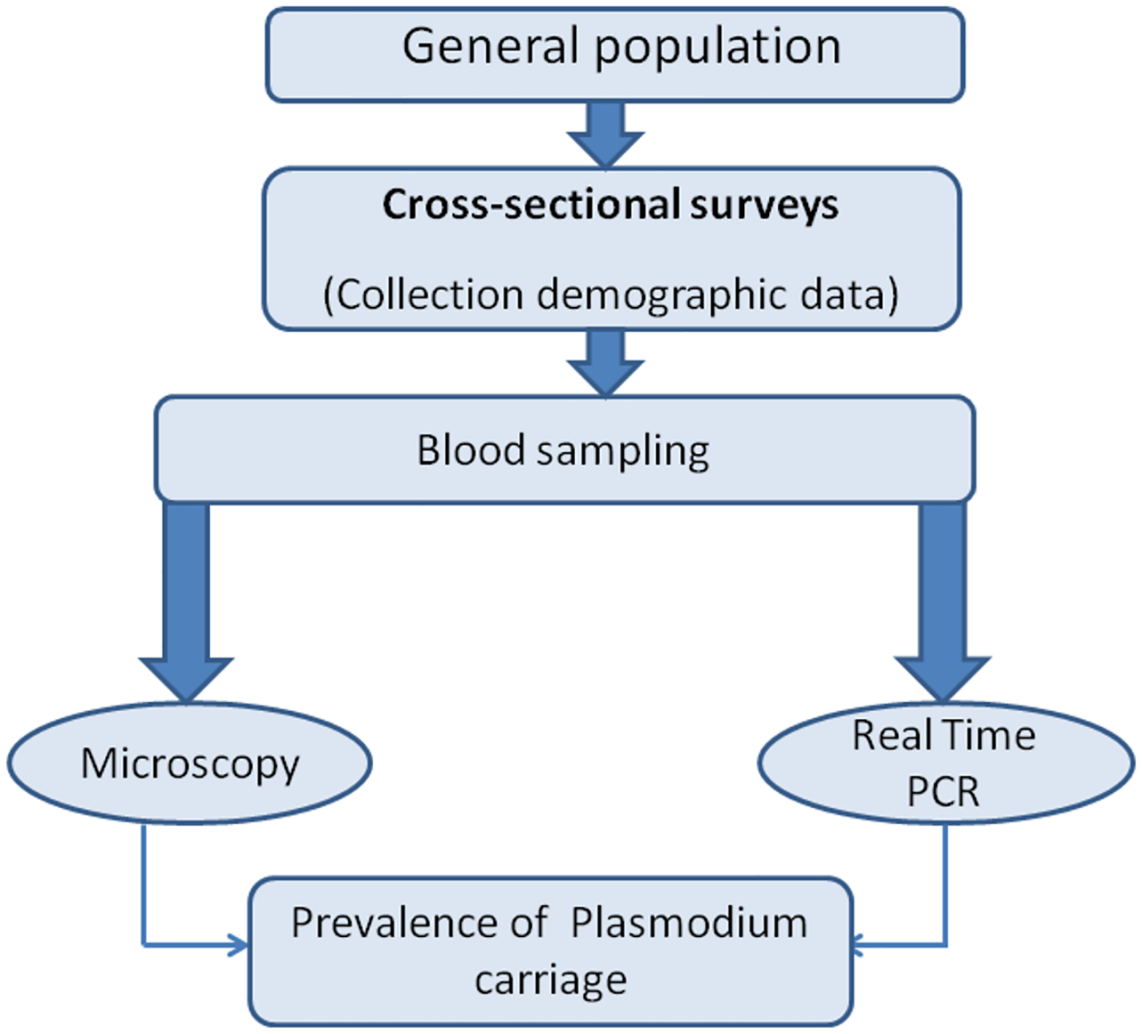
Study design for determination of *Plasmodium* carriage in Dielmo and Ndiop villages.

Community-based cross sectional surveys were conducted in both villages on July 2013, June 2014 and June 2015 respectively corresponding to periods preceding the malaria transmission season in Senegal. No formal sample size calculation was performed, as the objectives were to sample the largest possible fraction of the population involved in the project. A total of 2,037 blood samples were collected from asymptomatic individuals for determination of *Plasmodium* carriage by LM and quantitative Real Time PCR (qPCR) (Fig 1).

The study falls within the ongoing Dielmo/Ndiop project, a longitudinal prospective study of malaria infection and the determinants of the disease in a community that has been conducted since early 1990s. The study objectives, benefits and risks were explained in Serere and wolof (the most commonly spoken languages in the areas) or French (the official Senegalese language) to adults participants and legal guardians of minors before inclusion. Written informed consent was obtained from adults and legal guardians of minors participants. The study was examined and approved by the Senegalese National Health Research Ethics Committee.

### Microscopic examination of blood films

For microscopy determination of malaria parasites, thin and thick blood smears were prepared, air-dried, fixed in methanol, stained for 20–30 minutes in Giemsa and microscopically examined for malaria parasites by experienced microscopists at the Institute for Research and Development. At least two hundred oil-immersion fields were examined at 100X magnification before a slide was considered negative.

### Molecular detection and characterization of *Plasmodium* species

The detection of *Plasmodium* spp was carried out by qPCR following genomic DNA isolation of *Plasmodium* parasites using QIAamp DNA Blood Mini Kit (Qiagen, Hilden, Germany) according to manufacturer’s instructions. DNA from blood samples of known microscopically-confirmed *P*. *falciparum*, *P*. *malariae*, *P*. *ovale* and *P*. *vivax* infected patients served for positive controls in all amplifications; sterile water and uninfected samples served for negative control to ensure lack of contamination [23].

The qPCR-based detection and characterization of *Plasmodium* spp was based on two steps real time PCR as previously described [24, 25]. In the first step, *Plasmodium* parasites were detected by a “screening real-time PCR” with genus-specific primers targeting the *Plasmodium* Cytochrome B gene using the Eva Green dye (Solis Biodyne) followed by melt curve analysis of resulting amplicons. Amplification was conducted in a 20 μl reaction mixture containing 5 μl of genomic DNA, 4 μl of 5X Evagreen qPCR Master Mix (Solis Biodyne) and 10 μM of each primer. Amplification was run in a Bio-Rad CFX96 real time thermal cycler using previously described conditions [24]. In the second step, positive Plasmodium DNA samples were ten-fold diluted and analyzed for malaria species using a nested real-time PCR assays with genus-specific primers (*P*. *falciparum*, *P*. *vivax*, *P*. *malariae*, and *P*. *ovale*). Primers sequences and real-time PCR conditions used to characterize *Plasmodium* species were as described by Canier et al. [24]. The presence of *P*. *knowlesi*, the fifth human malaria parasite [26] was not investigated in this study.

Submicroscopic *Plasmodium* carriage was defined as an infection with *Plasmodium* detected by qPCR but not by LM.

### Statistical analysis

Data were analyzed using R statistical software. Distribution of submicroscopic *Plasmodium* carriage was analyzed according to three defined age groups (≤5 years, [5–15 years] and >15 years), sampling period (2013 to 2015), and village of origin (Dielmo and Ndiop) taking into account entomological inoculation rate (EIR) determined as detailed elsewhere [18, 27]. Fisher's exact tests were performed for testing the null hypothesis that the frequencies of submicroscopic carriage in the three defined age groups are the same. Chi2 test was used to compare prevalence of submicroscopic *Plasmodium* carriage between villages and across sampling periods. Statistical significance was considered when P values were less than or equal to 0.05.

## Results

### Demographic characteristics of the study population

A total of 2,037 blood samples collected from Dielmo and Ndiop in 2013 (N = 612), 2014 (N = 723) and 2015 (N = 702) were investigated in this study for the presence of *Plasmodium* parasites (Table 1). Female participants were predominant over males for all three sampling years (59.31% vs 40.69% in 2013, 54.08% vs 45.92% in 2014 and 53.70% vs 46.30% in 2015) (Table 1). Participants were in majority older than 15 years (48.52% in 2013, 52.69% in 2014 and 53.41% in 2015). The mean age of participant was 21.24 years (±19.29) in 2013, 14.82 years (±18.53) in 2014 and 22.54 years (±18.98) in 2015 (Table 1).

**Table 1:** Demographic characteristics of the study population.

Total	2013 (N = 612)		2014 (N = 723)		2015 (N = 702)	
	N	%	N	%	N	%
**Location**						
Dielmo	301	49.18	355	49.11	329	46.86
Ndiop	311	50.82	368	50.89	373	53.13
**Sex**						
Male	249	40.69	332	45.92	325	46.30
Female	363	59.31	391	54.08	377	53.70
**Age**						
Mean	21.24	**–**	14.82	**–**	22.54	**–**
Range	[0.4–91.5]	**–**	[0.2–92.5]	**–**	[0.2–93.4]	**–**
±SD	19.29		18.53		18.98	
**Age groups**						
≤ 5 years	113	18.46	121	16.73	112	15.95
[5–15] years	202	33	221	30.56	215	30.62
>15 years	297	48.52	381	52.69	375	53.41

### Validation of the qPCR assay for diagnosis of *Plasmodium* carriage

**Fig 2:**
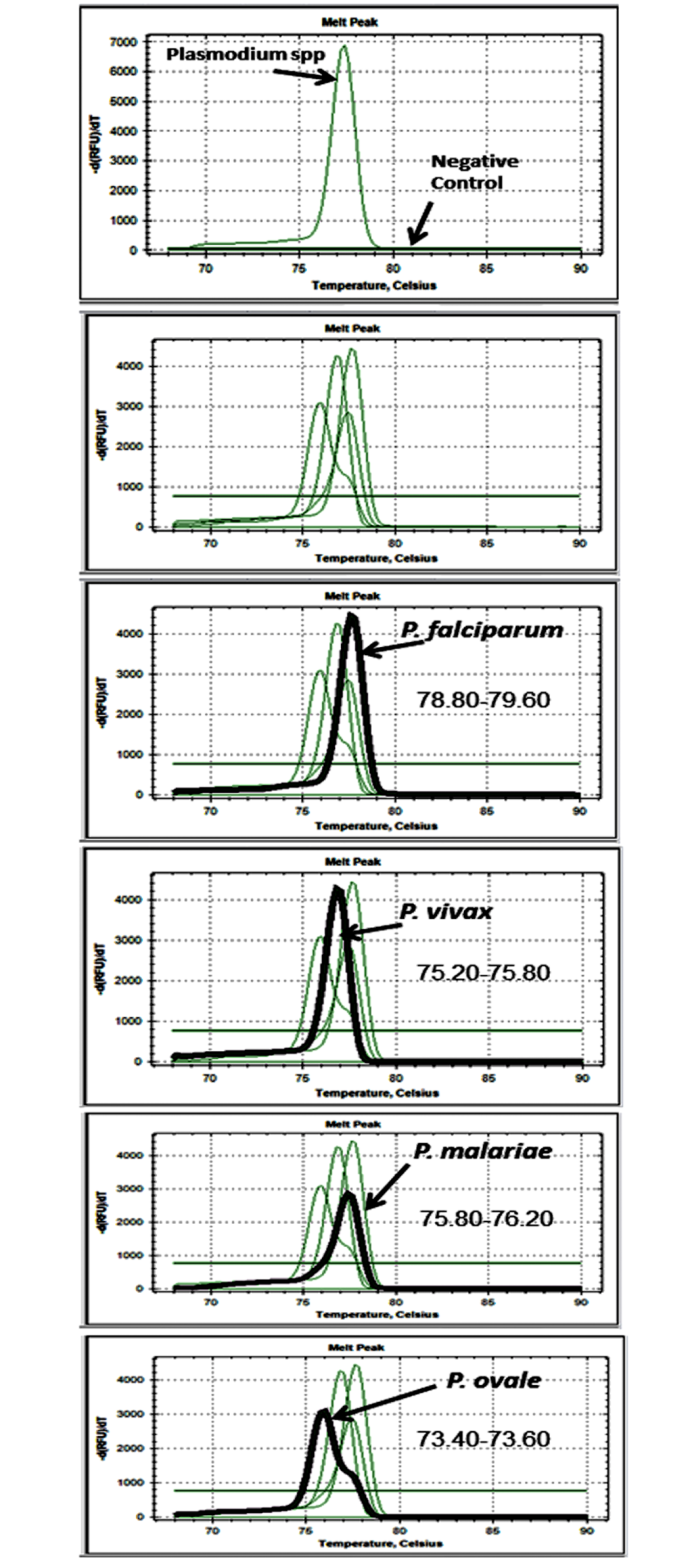
Melt curves analysis showing the qualitative detection and discrimination of the four malaria species (*P*. *falciparum*, *P*. *vivax*, *P*. *malariae* and *P*. *ovale*) using nested real time PCR.

To validate the qPCR-based assay for diagnosis and characterization of *Plasmodium* species, DNA obtained from known microscopy and nested PCR-confirmed *P*. *falciparum*, *P*. *vivax*, *P*. *malariae* and *P*. *ovale* infected samples were used as positive controls [23], sterile distilled water served for negative control (Fig 2). In a valid run, a positive amplification of *Plasmodium* DNA results in a melt curve appearing with a melt peak temperature between 76.20°C and 78.80°C (Fig 2A). The species-specific qPCR assays showed specific amplification with distinguishable melting curves associated with specific melt temperatures characteristics of each *Plasmodium* species (Fig 2B). Detailed analysis of the amplification and melt curves distinguished positive DNA amplification from *P*. *falciparum* (Fig 2C), *P*. *vivax* (Fig 2D), *P*. *malariae* (Fig 2E) and *P*. *ovale* (Fig 2F).

### Microscopy and qPCR-based prevalence of *Plasmodium* carriage

In 2013, no *Plasmodium* carriage was detected by microscopy among the 612 blood samples screened in Dielmo and Ndiop (Table 2) while qPCR-based screening detected *Plasmodium* DNA (submicroscopic *Plasmodium* carriage) in 4.65% (14/301) and 2.89% (9/311) of samples from Dielmo and Ndiop, respectively (Table 2). In 2014, LM examination of Giemsa-stained smears identified only 2 *Plasmodium* positive samples (1 in Dielmo and 1 in Ndiop) among the 723 samples screened representing a prevalence of 0.28% (1/355) and 0.27% (1/368) in Dielmo and Ndiop, respectively. The 2-microsocopy positive samples originated from a 9.7 years old male from Dielmo and a 0.6 years old female from Ndiop. The qPCR-based molecular diagnostic successfully amplified *Plasmodium* DNA in 12.72% (92/723) of samples collected in 2014 of which 6.22% (45/723) originated from Dielmo and 6.50% (47/723) from Ndiop (Table 2). The specific prevalence of *Plasmodium* carriage in Dielmo and Ndiop were 12.67% (45/355) and 12.77% (47/368) respectively (Table 2). In 2015, 702 giemsa-stained slides were screened for *Plasmodium* species (Table 2) and microscopic investigation revealed only 1 *Plasmodium* positive slide in Dielmo originating from a 16.6 years old male. By contrast, qPCR identified substantial *Plasmodium* carriage in both Dielmo (6.90%, 25/329) and Ndiop (5.44%, 21/373) among the corresponding 702 samples with available LM data. Sub-microscopic *Plasmodium* carriage represented 6.41% (45/702) of the overall tested samples with available microscopic data in 2015. All microscopy-positive samples from 2014 and 2015 sampling were also positive by qPCR.

**Table 2:** Prevalence of Plasmodium carriage based on microscopy and qPCR.

	2013 N = 612		2014 N = 723		2015 N = 702	
**Villages**	Dielmo	Ndiop	Dielmo	Ndiop	Dielmo	Ndiop
(N)	301	311	355	368	329	373
**EIR**	43	15	18	0	3	0
**Microscopy**						
N (%)	0 (0)	0 (0)	1 (0.28)	1 (0.27)	1 (0.30)	0 (0)
**qPCR**						
N (%)	14 (4.65)	9 (2.89)	45 (12.67)	47 (12.77)	25 (6.90)	21 (5.44)
**Submicroscopy**						
N (%)	14 (4.65)	9 (2.89)	44 (12.39)	46 (12.5)	24 (7.29)	5.63)

### Details of sub-microscopic *Plasmodium* species

Single *P falciparum* infections accounted for the majority of submicroscopic *Plasmodium* carriage representing 86.95% (20/23), 87.77% (79/90) and 95.55% (43/45) of detected *Plasmodium* in 2013, 2014 and 2015 respectively (Table 3). Single *Plasmodium malariae* infections were present in 8.69% (2/23) and 2.22% (2/90) of sub-microscopic *Plasmodium* spp in 2013 and 2014 respectively while *P*. *malariae* was not detected in 2015 (Table 3). *Plasmodium ovale* was absent as single infection in both 2013 and 2014 sampling while being present as mixed infections with *P*. *falciparum* in 4.34% (1/23) and 10% (9/90) of sub-microscopic infections in 2013 and 2014, respectively (Table 3). By contrast, *P*. *ovale* was present in 2015 uniquely as single infections in 4.44% (2/45) of sub-microscopic infected samples (Table 3). No *P*. *vivax* was found from all three surveys in both villages.

**Table 3:** Details of submicroscopic *Plasmodium* species.

	2013 N = 612		2014 N = 723		2015 N = 702	
**Villages**	Dielmo	Ndiop	Dielmo	Ndiop	Dielmo	Ndiop
(N)	301	311	355	368	329	373
**Sub-microscopy**						
N (%)	14 (4.65)	9 (2.89)	44 (12.39)	46 (12.5)	24 (7.29)	21 (5.63)
***Plasmodium* spp**						
*P*. *falciparum*	13 (92.85)	7 (77.77)	40 (90.90)	39 (84.78)	23 (95,83)	20 (95.24)
*P*. *malariae*	1 (7.14)	1 (11.11)	2 (4.54)	0	0	0
*P*. *ovale*	0	0	0	0	1 (4.00)	1 (4.76)
*P*. *vivax*	0	0	0	0	0	0
Mixed P. f+P. o	0	1 (11.11)	2 (4.54)	7 (15.21)	0	0

### Submicroscopic *Plasmodium* carriage according to age, village and sampling period

**Fig 3:**
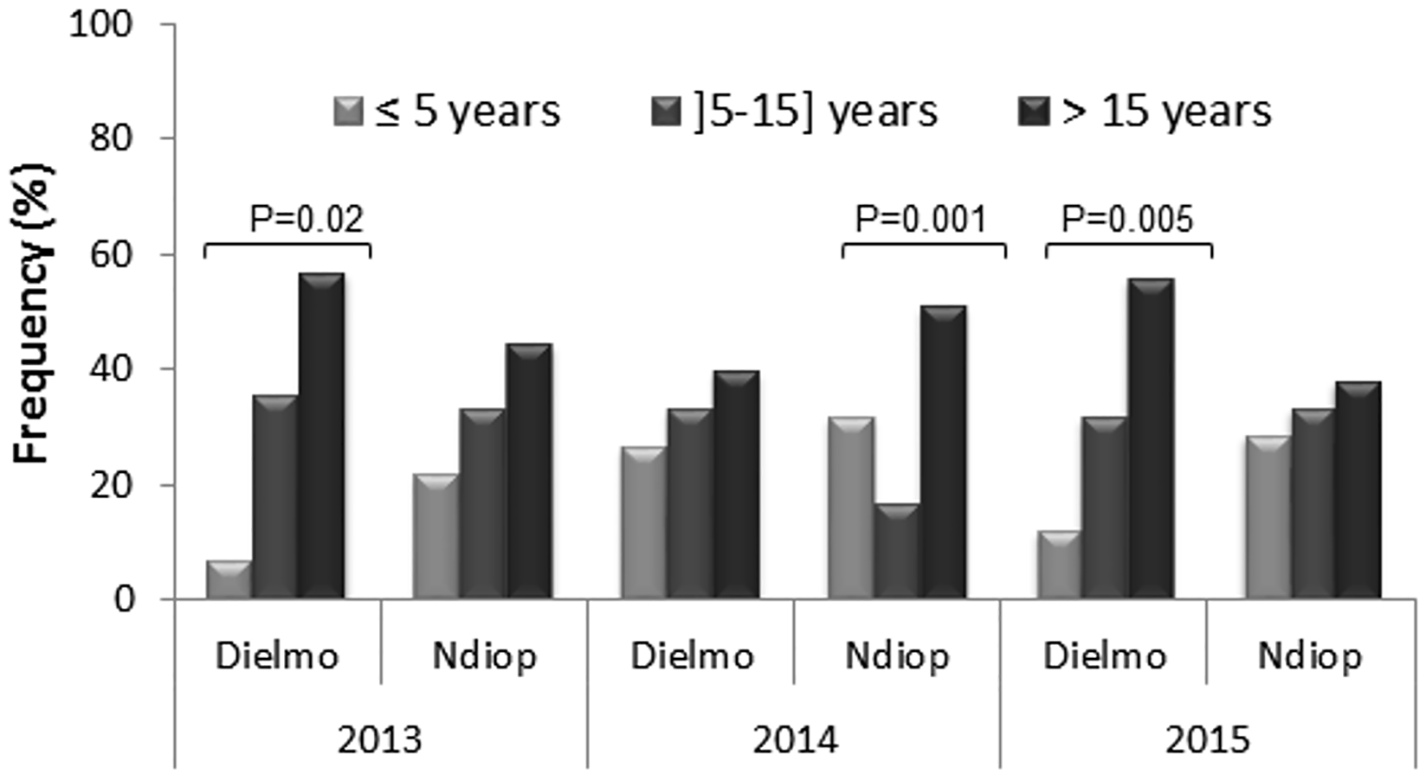
Frequency of submicroscopic *Plasmodium* parasite carriage according to age groups.

The carriage of submicroscopic *Plasmodium* spp was compared between age groups (≤ 5, [5–15] and > 15 years), villages (Dielmo and Ndiop) and sampling period (2013 to 2014). Submicroscopic *Plasmodium* carriage was similarly distributed across the three-years sampling periods (2013, 2014 and 2015) and in both villages (Dielmo and Ndiop) and was characterized by an age-dependent carriage of *Plasmodium* parasites with frequencies being highest in older individuals (> 15 years) and lowest in younger ones (≤ 5 years) (Fig 3). The unique exception to this overall observed distribution was the significantly higher frequency of submicroscopic carriage in infants (≤ 5 years) over younger adults [5–15 years] observed in Ndiop in 2014 (Fig 3). Statistical comparisons performed using Fisher’s exact tests showed that difference of frequencies between age groups were significant in Dielmo in 2013 (p = 0.02) and 2015 (p = 0.005) and in Ndiop in 2014 (p = 0.001).

With respect to village of origin of samples, submicroscopic *Plasmodium* carriage was not significantly different between Dielmo and Ndiop in 2013 (X^2^ = 0.86, p = 0.35), 2014 (X^2^ = 3.6x10^-31^, p = 1) and 2015 (X^2^ = 0.55, p = 046) despite variation of transmission as determined by the annual entomological inoculate rate (EIR) (Table 2). By contrast, submicroscopic *Plasmodium* carriage differs significantly across the three-year sampling periods both in Dielmo (X^2^ = 13.99, p = 0.001) and Ndiop (X^2^ = 1.6x10^-6^, p<0.001), similar to the EIR (Table 2).

## Discussion

Given the renewed focus on continuous reduction of malaria transmission, morbidity and mortality in many endemic countries ultimately leading to elimination of the disease, malaria control efforts deserve to focus on *Plasmodium* infection (rather than morbidity and mortality) to reduce and subsequently suppress the reservoir. The function “reservoir” is multifactorial and involves humans (but also non-human primates), vectors and Plasmodia. From the parasite perspective, the rapid and accurate diagnostic of low-density submicroscopic infections that are usually missed by conventional RDT and LM diagnostic methods remains a challenge. In fact, despite their many advantages, the limitations and shortcomings of both RDT and LM, to detect low-density parasitaemia are well documented elsewhere [5, 9]. The introduction of sensitive molecular detection techniques that amplify parasite DNA, has considerably improved the performance of malaria diagnostic and is increasingly revealing the widespread presence of malaria parasite infections with densities below the detection threshold of RDT and LM [8, 9], thus fundamentally changing the current view of malaria epidemiology and burden of infection.

In the present study, a reliable and cost effective molecular-based malaria diagnostic targeting the *Plasmodium cytochrome b* gene [24] was used to detect submicroscopic *Plasmodium* carriage in Dielmo and Ndiop, two malaria endemic villages where all malaria indicators have declined substantially over the last decade. The study revealed substantial submicroscopic *Plasmodium* carriage among asymptomatic individuals at various proportions in both villages prior the malaria transmission season. These findings are important as they provide a basis for identifying and defining priority areas for control interventions when elimination strategies are being considered.

The report of similar findings from others studies conducted in different malaria transmission contexts [4, 5, 28, 29] highlights the need for massive deployment of molecular diagnostics to target low-density submicroscopic parasites which may represent a significant challenge to malaria elimination efforts. Real time PCR has also a limit of detection as reported in many studies [24, 25] implying that the number of submicroscopic infections is likely higher than reported in this study. It is apparent that LM underestimated the true parasite carriage within the study population as it detected none of the infections identified by qPCR in 2013 while being able to detect only 0.27% and 0.14% of *Plasmodium* carriage in 2014 and 2015, respectively compared to 12.72% and 6.01% prevalence revealed by qPCR.

In this study, submicroscopic *Plasmodium* carriage was age-dependent, similar to reports from others studies that have shown that individuals older than 15 years were several times more likely to carry submicroscopic parasites than younger individuals, thus arguing for a link between increasing age and lower parasite densities [6, 30]. The finding may explain the fact that infections will be controlled and remain asymptomatic in older individuals because clinical immunity develops overtime. However, studies of factors such as host and parasite genetics that are potentially involved in the observed epidemiological situation, would tremendously contribute to unravel the determinants of submicroscopic *Plasmodium* carriage in a community.

The absence of significant difference in the prevalence of submicroscopic *Plasmodium* carriage observed between the two studied villages despite the difference in malaria transmission intensity, could suggest that submicroscopic *Plasmodium* carriage is not related to malaria transmission intensity. This is supported by the fact that the significant difference of prevalence observed across sampling period (2013 to 2015) both in Dielmo and Ndiop was unrelated to the malaria transmission intensity.

The higher prevalence of submicroscopic *P*. *falciparum* carriage in the study areas may have important implications for malaria control measures in Senegal since such infections may be important contributors to the infectious reservoir as documented elsewhere [15, 31, 32]. In fact, it has been reported that, even at low densities, submicroscopic infections could be a potential source of transmission for vectors and a potential source of malaria attack within the population when adequate transmission conditions are established [33, 34]. The relative contribution of submicroscopic infections for onward malaria transmission in low endemic settings is still under debate despite findings from molecular-based surveys that have shown that submicroscopic carriers are presumed to be the source of 20–50% of all human-to-mosquito transmissions [35, 36].

From a clinical point of view, low-density infection may have significant public health importance, as studies conducted by Bousema and Drakeley have shown that mosquitoes feeding on LM parasite-negative individuals can become infected with malaria [37]. From the perspective of control programme aiming to further reduce malaria transmission and achieve elimination, the important question of the contribution of submicroscopic parasite carriage in sustaining transmission remains to be addressed. As described in detail previously [15, 36], there is a considerable probability of transmission occurring at parasite densities often missed by LM and RDT according to both mathematical theory and data.

Further studies to unveil the magnitude of low-density submicroscopic malaria parasites carriage at the country level both within asymptomatic and symptomatic individuals coupled with entomological investigations addressing their role in sustaining malaria transmission are needed to guide accelerated efforts towards malaria elimination in Senegal.

## Conclusion

The community-based surveys conducted prior the malaria transmission season in Ndiop and Dielmo revealed substantial submicroscopic *Plasmodium* carriage among asymptomatic individuals, thus indicating that estimates based on routinely used diagnostic techniques such as RDT and LM, grossly underestimate true malaria parasite prevalence. The findings reported in this study warn against a relaxation of control efforts once prevalence of infection appears low according to LM. The results have also important practical implication for potential malaria elimination strategies in the areas as they could guide a mass screening and treatment approach to accompany elimination efforts in the areas that can be subsequently extended countrywide.
